# Hybrid Approach to Enabling Cross-Domain Service Orchestration over Heterogeneous Infrastructures

**DOI:** 10.3390/s25185804

**Published:** 2025-09-17

**Authors:** Jane Frances Pajo, Geir Egeland, Sarang Kahvazadeh, Hamzeh Khalili, Martin Tolan, Ryan McCloskey, Min Xie, Olai Bendik Erdal

**Affiliations:** 1 Telenor Research & Innovation, Telenor ASA, 1360 Fornebu, Norway; 2 Centre Tecnològic Telecomunicacions Catalunya (CTTC), 08860 Castelldefels, Spain; 3 Walton Institute, South East Technological University, X91 P20H Waterford, Ireland

**Keywords:** cloud-native, ETSI OSM, multi-domain, netApps, orchestration

## Abstract

Efforts to lower the barriers for 5G uptake have brought forth accelerated innovation rates in a wide range of verticals. Consequently, the demands for 5G experimentation facilities have recently emerged from small- and medium-sized enterprises (SMEs) and 3rd party developers, requiring the abstraction of the complexities of the underlying infrastructure, platform, and related management and orchestration (MANO) systems, especially in multi-domain scenarios. This paper proposes a novel approach towards cross-domain service orchestration, which combines the flexibility of supporting different 5G Service Orchestrators (SOs) in various domains, while preserving compatibility through NetApps. The Cross-domain Service Orchestrator (CDSO) is based on ETSI’s Open Source MANO (OSM), with a Requests Handler module on top, which acts as the main integration point to the different domains’ 5G SOs and other custom systems that are northbound. This would facilitate the interworking among independently orchestrated domains in supporting multi-domain services. The EU Horizon 2020 project, 5GMediaHUB, is presented as a use case, together with a first implementation of the Requests Handler and corresponding integrations. Nonetheless, the proposed approach is vertical-agnostic and is foreseen to accelerate service innovation and digitalization in any industry by laying the foundations in terms of service management and interconnectivity.

## 1. Introduction

Europe’s Digital Single Market Strategy [1] seeks to tear down the barriers for small- and medium-sized enterprises (SMEs) and 3rd party developers in relation to digitalization. Various 5G and beyond 5G (B5G) technologies are expected to enable more-disruptive applications and services from various vertical markets, boosting innovation and prototyping. Such verticals come with highly heterogeneous requirements, in terms of both quality of service (QoS) and quality of experience (QoE), and could potentially span multiple administrative and technological domains. Hence, proving success with 5G/B5G vertical trials entails handling the management complexities that come with such heterogeneity.

Pan-European pre-commercial testbeds have also gained popularity from 5G-PPP projects like 5G-VINNI [2] and 5G-EVE [3], among others, for fostering vertical innovation and extensive trials. They have proven to advance 5G trials in a wide range of verticals (e.g., eHealth, Transportation, Industry 4.0, etc.), as well as in various use case scenarios (potentially spanning multiple domains). The business case for such experimentation facilities has also been recognized, along with a multi-stakeholder ecosystem [4].

Moreover, with the current demand for self-service platforms on which vertical stakeholders could carry out development, testing, and/or performance evaluation on demand, it has become fundamental to abstract the underlying complexities of (multi-domain) infrastructures/networks and slice/service lifecycle management from the platform users.

Two key enablers have been identified for such an abstraction: (i) **NetApps** and (ii) a **Cross-domain Service Orchestrator (CDSO)**. NetApps are functions that abstract vertical applications from Network Services (NSs). They can be thought of as being akin to NSs in terms of packaging (with physical, virtual, and/or containerized constituent components) and management, but offer value-added services (either specific or agnostic to verticals). The CDSO will manage the lifecycle of NetApps across involved domains, while taking care of the attachment points between NetApps and 5G NSs. Note that an end-to-end (E2E) service can involve multiple NetApps, and having a single pane of glass for NetApp management on the CDSO (regardless of which domain a NetApp is deployed) would definitely lower the operation complexity.

With this in mind, a CDSO based on ETSI’s Open Source MANO (OSM) [5] is presented in this paper. The CDSO internal architecture is presented with a Requests Handler module on top of the OSM, which is designed as the main integration point in relation to the different components of the E2E chain. Additionally, workflows for (i) NetApp onboarding and (ii) E2E slice ordering are illustrated and described. Though originally proposed under the framework of 5GMediaHUB [6], the approach is expected to go beyond the project, i.e., any Custom System northbound can be integrated into the CDSO, and benefit from a multi-domain platform. The 5GMediaHUB use case is presented as a proof-of-concept, with a first implementation of the Requests Handler and the NetApp onboarding workflow. In this case, the Custom System is 5GMediaHUB’s *Experimentation Tools* layer, which enable developers to design, onboard, and test media NetApps and next-generation scenarios.

The remainder of this paper is organized as follows. Section 2 provides a brief background and related work on cross-domain service orchestration. Section 3 takes a closer look into the proposed approach and CDSO internal design, followed by the related workflows in Section 4. Section 5 describes the 5GMediaHUB use case with implementation and integration perspectives. Finally, conclusions are drawn in Section 6.

## 2. Background and Related Work

The cross-domain orchestration landscape for E2E services is shaped by varying interpretations from different organizations and standardization bodies with respect to key concepts like slices, services, and network services [7]. Consequently, a number of 5G-PPP projects adopted differing paths in the development of cross-domain federation models.

For example, the 5G-VINNI project adopts a federated, standards-based approach to orchestrate network slices across domains, enhancing interoperability and service delivery [8]. However, this approach introduces operational complexity when coordinating multiple orchestration systems across large networks. Similarly, the 5G-EPICENTRE project [9] focuses on NetApp-based development, federating testbeds through a Karmada control plane. However, it is limited to Kubernetes, overlooking Network Function Virtualization (NFV) architectures. Several projects, like 5GCity [10] and 5GTango [11], have contributed to cross-domain orchestration, primarily within NFV-compliant, single-domain environments. These projects, while valuable, highlight the need for orchestration solutions that transcend single-domain, Kubernetes-only or NFV-only setups to support multi-domain service delivery.

Other works in the literature have also explored different aspects of federation, including combinations of federation models such as hierarchical, peer-to-peer, and recursive configurations but also involving the deployment of specific MANO artifacts in each domain and federation point [12], as well as the use of marketplace and auction strategies towards cooperative resource provisioning across the domains [13].

Note that the term ‘domain’ has been used in different contexts in the literature, such as technological domains of the radio access, transport and core network segments, and telecom operators’ administrative domains. In this work, ‘domain’ refers to an administrative boundary, such as a public or private mobile network, each with distinct network identities. We propose an approach that addresses interoperability challenges observed in existing projects, paving the way for more flexible and scalable cross-domain orchestration.

The following sub-sections seek to provide a mapping of the 3GPP-ETSI slice management framework with the federation models adopted in different 5G-PPP projects, highlighting the novelty of the proposed approach.

### 2.1. Slice Management in 3GPP-ETSI

In 3GPP [14], three main functions are associated with network slice lifecycle management and orchestration (MANO), as follows:

- Communication Service Management Function (CSMF): Translates service-related requirements to network slice specifications.
- Network Slice Management Function (NSMF): Manages E2E slices and derives network slice subnet requirements.
- Network Slice Subnet Management Function (NSSMF): Manages individual network slice subnets.

These functions are grouped in the *Vertical Slicer* layer, where service requirements from verticals are mapped to network slices. Each network slice (or subnet) is further decomposed into network services, which are broken down into physical/virtual/containerized functions (PNFs/VNFs/CNFs), managed within ETSI’s NFV-MANO framework [15].

### 2.2. Federation Models Adopted in 5G-PPP Projects

**Figure 1:**
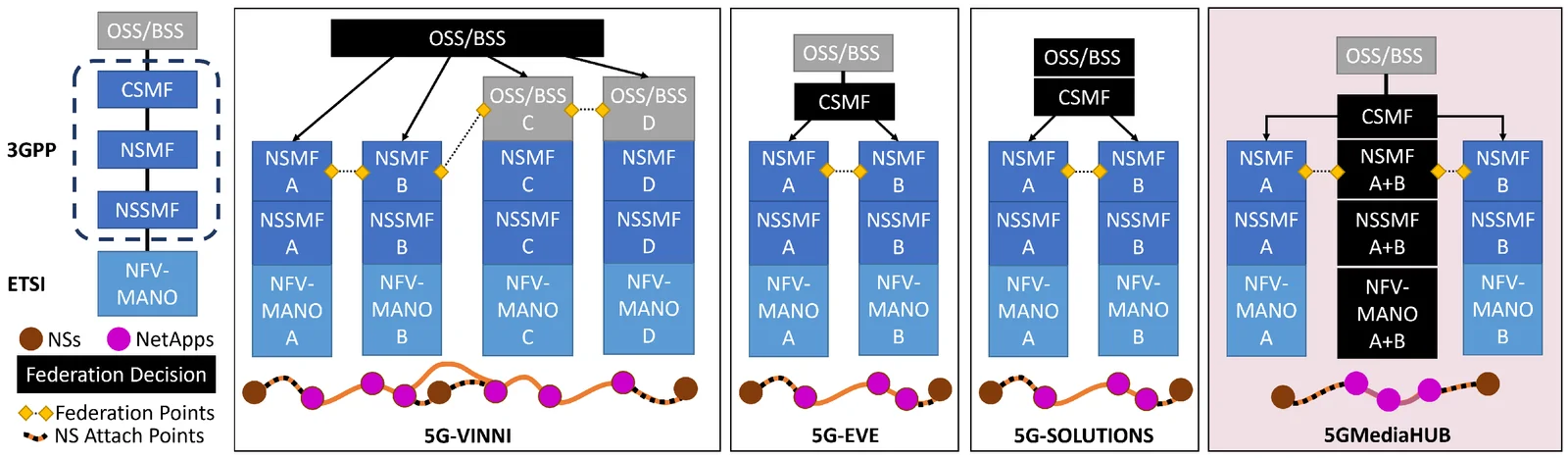
Comparison of federation models adopted in 5G-PPP projects and their mapping to 3GPP.

In single-domain scenarios, vertical slice requests are processed through the Operations Support System / Business Support System (OSS/BSS) layer and are mapped to resources via the 3GPP-ETSI chain. Multi-domain scenarios, however, require federation across these components, a challenge that is addressed by various 5G-PPP projects. As shown in Figure 1, federation decisions in these projects occur at different levels: (i) **OSS/BSS** for 5G-VINNI’s OSS; (ii) **CSMF** for 5G-EVE’s Multi-site Network Service Orchestrator; (iii) **OSS/BSS+CSMF** for 5G-SOLUTIONS’ CDSO [16]; and (iv) the **full 3GPP-ETSI stack** for 5GMediaHUB’s CDSO. Although the proposed approach appears complex, it simplifies operations by using OSM to consolidate these roles, with the CDSO managing NetApp lifecycles and 5G SOs handling 5G NSs.

As regards federation points, three types can be seen from Figure 1: (i) **OSS/BSS-to-OSS/BSS**; (ii) **OSS/BSS-to-NSMF**; and (iii) **NSMF-to-NSMF**. The 5G-VINNI system had ambitions to experiment with all three types, and the second type (i.e., interworking between OSS/BSS and NSMF) proved to be the most challenging. The third type is common among all projects, but 5GMediaHUB’s novelty comes with the CDSO having its own NSMF, which needs to interwork with each of the domains’ NSMFs. Due to the aforementioned separation of concerns, federation is further simplified as the **NSMF-to-NSMF** interaction, which will only have to handle the attach points between the 5G NSs and NetApps; the chaining of NetApps will be handled by the CDSO itself. Hence, from a potential fully meshed to a star setup, the domains will now only need to integrate with the CDSO, improving scalability.

In particular, each domain needs to expose (i) Virtual Infrastructure Manager/s (VIM/s) and/or Container Infrastructure Service Manager/s (CISMs) for hosting the NetApps, and (ii) 5G SO for 5G NS ordering. By giving the domains the flexibility and control to host platform/s and 5G NSs, which are to be made available for cross-domain services, and having a single pane of glass for NetApp management on the CDSO, potential conflicts inherent to multi-stakeholder environments can be mitigated and minimized. OSM has long been envisioned to interwork with independently orchestrated domains—either by a proprietary orchestration system or by another OSM instance—in a layered architecture [17], matching the design principles of the CDSO.

The CDSO of 5GMediaHUB is foreseen to go beyond the project in terms of enabling interworking among independently orchestrated 5G domains, which is now becoming a must-have for supporting 5G and beyond use cases and services.

## 3. Cross-Domain Service Orchestration

An overview of the proposed approach is first presented in this section, followed by an in-depth description of the CDSO’s internal architecture. Moreover, the Platform-as-a-Service (PaaS) NetApp is presented as a supplementary CDSO feature—a cornerstone of its unique approach of combining NFV-compliant (for OSM deployment) and cloud-native (for standard Kubernetes deployments) NetApps.

### 3.1. Proposed Approach

As services start to span multiple sites and/or multiple operator domains—each potentially managed by its own orchestration system—it is necessary to enable both data plane connectivity among sites and interworking among their orchestration systems. The proposed federation model is unique to this end. For instance, the right-most part of Figure 1 illustrates a cross-domain E2E slice, involving two domains—Domain A and Domain B. User equipment (UE) in each domain can connect to their corresponding 5G NSs, which are then attached to the NetApps. A domain’s 5G SO is responsible for the lifecycle management of the 5G NSs (e.g., of the 5G Core). On the other hand, the CDSO is responsible for the lifecycle management of the NetApps, as well as the constituent functions in the chain, which are potentially deployed in multiple domains. Additionally, the CDSO needs to coordinate with the involved domains’ 5G SOs to forward the necessary information for attaching NetApps to the 5G NSs, as well as attaching the latter to UEs.

Each domain is supposed to expose one or more VIM/s and/or CISMs to the CDSO, on which the NetApps can be deployed. It is important to note that both 5G NSs and NetApps can be mapped to a 3GPP Network Slice Subnet Instance (NSSI), and one or several NetApps can be deployed in any domain(s) as part of a use case scenario. This means that the E2E slice will be composed of 5G NSs and NetApps (from one or several NetApps) deployed in the involved domain(s).

### 3.2. Internal Architecture

OSM is used as a basis for the CDSO, with its well-established Information Model (IM), Northbound Interface (NBI), multi-site deployment support, and monitoring capabilities. OSM already supports VIM/CISM application programming interfaces (APIs) for the lifecycle management and monitoring of NSs (and/or NetApps) on a wide selection of VIM/CISM targets (e.g., Openstack VIMs and Kubernetes clusters, among others). To the best of our knowledge, previous OSM-based federation focused on multi-site deployments, in which a centralized orchestrator manages the lifecycle of services (both 3GPP and non-3GPP alike) for all domains.

However, it is important to take into account that the domains may adopt different technologies and architectures across their infrastructures. What was missing was the ability to enable the domains to independently orchestrate their respective 5G networks, in potentially different approaches (e.g., an open-source 5G SO or a proprietary one).

To bridge this gap, a Requests Handler module is designed on top of OSM to handle the necessary integrations, specifically to translate/forward the different requests from any Custom System northbound (e.g., at the OSS/BSS level) towards the CDSO’s OSM instance and towards the domains’ 5G SOs. The Requests Handler seeks to be the main integration point that facilitates interworking among independently orchestrated 5G domains.

**Figure 2:**
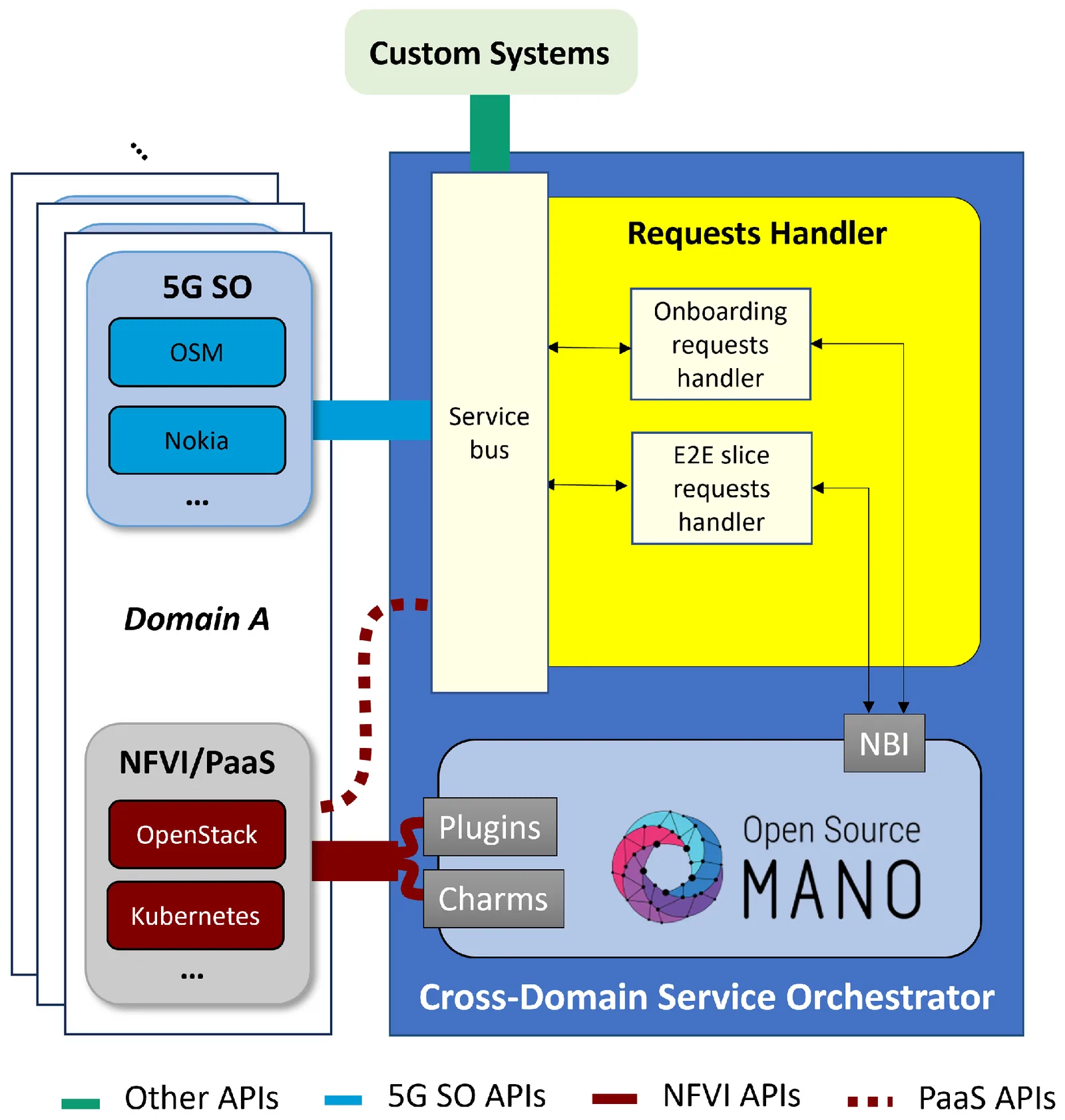
Internal architecture of an OSM-based CDSO.

As shown in Figure 2, the Requests Handler includes the following three components:

- The Service bus, which is the CDSO’s main interface with the Custom Systems, as well as with the domains’ 5G SOs. It primarily acts as the hub for all requests/response messages to/from the CDSO.
- The onboarding Requests Handler, which receives the onboarding requests from the Custom Systems for customized NetApps, then uploads the corresponding NF/NS packages to the CDSO’s OSM instance, or directly to a given PaaS NetApp instance.
- The E2E slice Requests Handler, which is in charge of translating the E2E slice requests into subslice requests for the CDSO’s OSM instance (for the NetApps) and the domains’ 5G SOs (for the 5G NSs), including the subslices’ and UE attachments.

### 3.3. The PaaS NetApp

PaaS serves as an abstraction layer for NetApps, aligning with cloud-native design principles. This layer streamlines the use of container technologies while abstracting the underlying cloud infrastructure—including networking, servers, operating systems, and storage and platform services. This enables developers to focus on deploying and configuring applications without managing infrastructure complexity, as well as the need for packaging the NetApps into CNFs.

Within this architectural framework, the PaaS abstraction layer is delivered through a PaaS NetApp [18], which falls into the categories of either a VNF Common Service or a VNF Dedicated Service in accordance with ETSI GR NFV-IFA 029 [19]. Implemented as a container-based service, it operates atop the NFVI (specifically, OpenStack) and is realized through a set of Ansible scripts for the Kubespray controller, deployed by the OSM. These Ansible scripts automate the generation of cloud-init files, which bootstraps Kubernetes clusters hosted within VNFs instantiated by OSM. One VNF acts as the Kubernetes controller, and cluster scaling is achieved by instantiating or deleting VNF hosts. OSM orchestrates network resources, while the CISM (Kubernetes) within the VIM handles containerized workloads and services.

In this setup, the CDSO’s OSM can orchestrate and delete the VMs responsible for hosting Kubernetes clusters (network resources), while Kubernetes itself acts as the application orchestrator. The PaaS NetApp interfaces with OSM via REST APIs to create and launch a cluster as an NSSI. Additionally, it connects with the Kubernetes cluster through the RESTful API, utilizing Kubespray and the Kubernetes Federation API v2 as the PaaS controller.

## 4. Workflows

Workflows for NetApp onboarding and E2E slice ordering are described and illustrated in this section, capturing the different interactions between a Custom System, the CDSO internal components, and the domains’ 5G SOs.

The CDSO is expected to handle five types of requests involved in the following workflows. Particularly, an *Onboarding request* is generated by the Custom System each time a new NetApp needs to be onboarded to the CDSO, which contains the NF and NS packages involved either for NFV-compliant or cloud-native NetApps. In the case of experimentations, a *UE specification* may also be involved, providing details on the UE type and location/density attachment information (e.g., IMSI, etc.) necessary to connect the UE to the 5G network. It will be included in the *E2E slice request* that will be sent to the CDSO, along with the details on the NetApps and 5G NSs to be used, as well as the E2E Service-level Agreements/Specifications (SLAs/SLSs).

The CDSO’s Requests Handler module will then translate the latter into (i) the *NetApp subslice request(s)* towards the internal OSM instance for (multi-site) NetApp deployment, and (ii) the *5G NSs subslice request(s)* towards the 5G SO(s) of the involved domains for 5G connectivity. The requests should also include a translation of the E2E SLAs/SLSs to per-subslice SLSs, which would become the bases for lifecycle management policies (e.g., autoscaling, migration, etc.).

### 4.1. NetApp Onboarding

**Figure 3:**
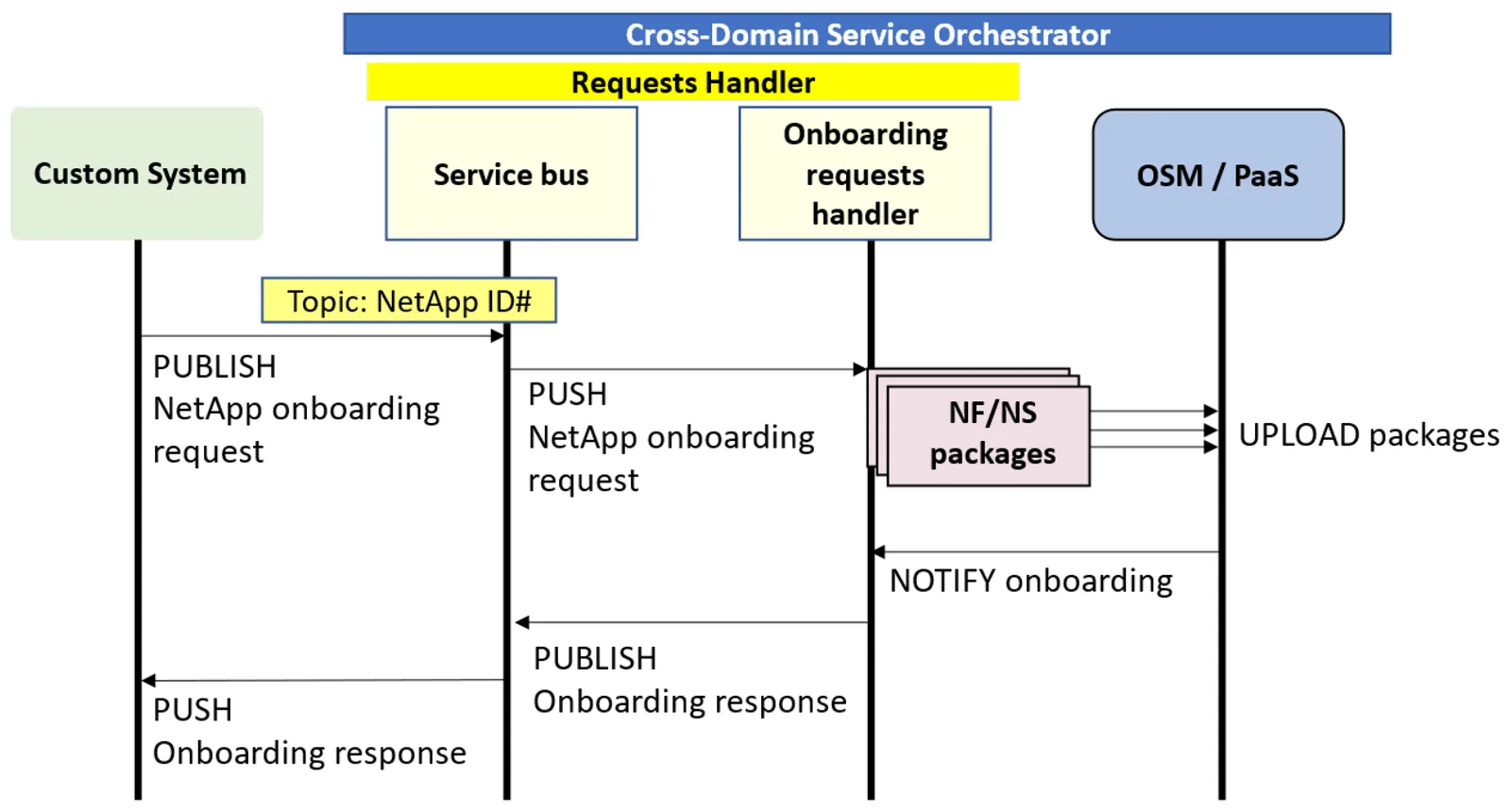
NetApp onboarding workflow.

The NetApp onboarding workflow is illustrated in Figure 3. The first step is for the Custom System to publish a *NetApp onboarding request* to the Service bus of the CDSO’s Requests Handler, with the **NetApp ID#** as the topic. The Service bus will then push the request to the onboarding Requests Handler, which extracts the NF/NS packages from the request and uploads them to the CDSO’s OSM instance or a given PaaS NetApp instance. When the packages have been successfully onboarded, the onboarding Requests Handler will publish the onboarding response on the Service bus, which will push the response to the Custom System backend.

### 4.2. E2E Slice Ordering

**Figure 4:**
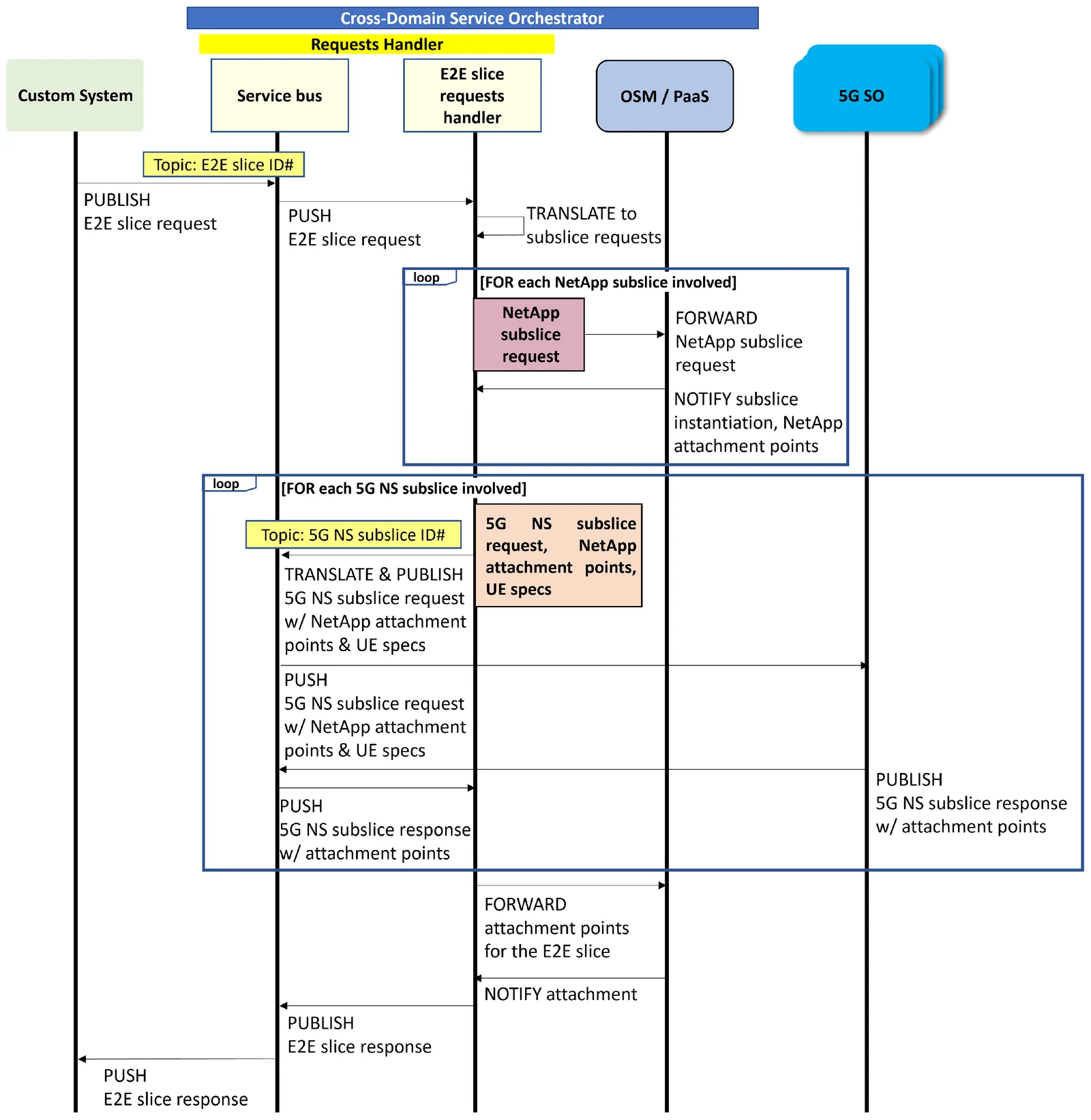
E2E slice ordering workflow.

The E2E slice ordering workflow is illustrated in Figure 4. The first step is for the Custom System to publish an *E2E slice request* to the Service bus of the CDSO’s Requests Handler, with the **E2E slice ID#** as the topic. The Service bus will then push the request to the E2E slice Requests Handler for translation.

Based on the required NetApp(s) and location information from the *UE specification*, the E2E slice Requests Handler generates the corresponding *NetApp subslice request(s)*, tagged with the VIM/CISM target(s) and SLSs. This is then forwarded to the CDSO’s OSM instance for instantiation. Once the NetApp(s) has/have been successfully instantiated, the E2E slice Requests Handler obtains the NetApp(s) attachment points among NetApp subslices relating to the 5G NSs. The E2E slice Requests Handler then generates the required *5G NS subslice request(s)*, which translates a domain-specific *UE subspecification* and indicates the NetApp(s) attachment points. Next, we publish the request(s) to the Service bus with their respective **5G NS subslice ID#**; then, we push them to the 5G SO(s) of the involved domain(s). Once the 5G NS(s) has/have been successfully instantiated, the 5G SO(s) will publish the response(s) to the Service bus, as well as indicating the 5G NS(s) attachment points in relation to the NetApp subslices. The Service bus will push them to the E2E slice Requests Handler. The latter will consolidate the attachment points information for the E2E slice and forward it to the CDSO’s OSM instance for configuration.

Once the subslices are successfully attached, the E2E slice Requests Handler will publish an E2E slice response to the Service bus; in turn, this will push the response to the Custom System backend.

While the concept of attachment points has been widely accepted, its realization is still far from formalization, as pointed out in [20]; hence, the proposed leveraging self-service and provider networks were realized for such attachment points. These can be used as starting points while also exploring other approaches for attachment point realization.

## 5. The 5GMediaHUB Use Case

The 5GMediaHUB model seeks to offer an elastic, secure, and multi-tenant 5G experimentation facility for the development, testing, and validation of NetApps for 3rd party media stakeholders. This will accelerate 5G technology uptake in the media industry by minimizing deployment ambiguities for 5G-empowered media applications and services.

Abstracting the underlying infrastructure- and management-level complexities is fundamental for platform usability and uptake by media stakeholders, especially for SMEs and 3rd party media application/NetApp developers. The CDSO is a key component, acting as an umbrella orchestrator that automates network/service and slice management across the domains of the underlying heterogeneous 5G testbeds. Two 5G testbeds, operated by Telenor (Norway) and CTTC (Spain), are interconnected over the GÈANT network and are made available for the facility, hence providing two domains for the use case scenarios and supporting multi-domain NetApp deployments.

**Figure 5:**
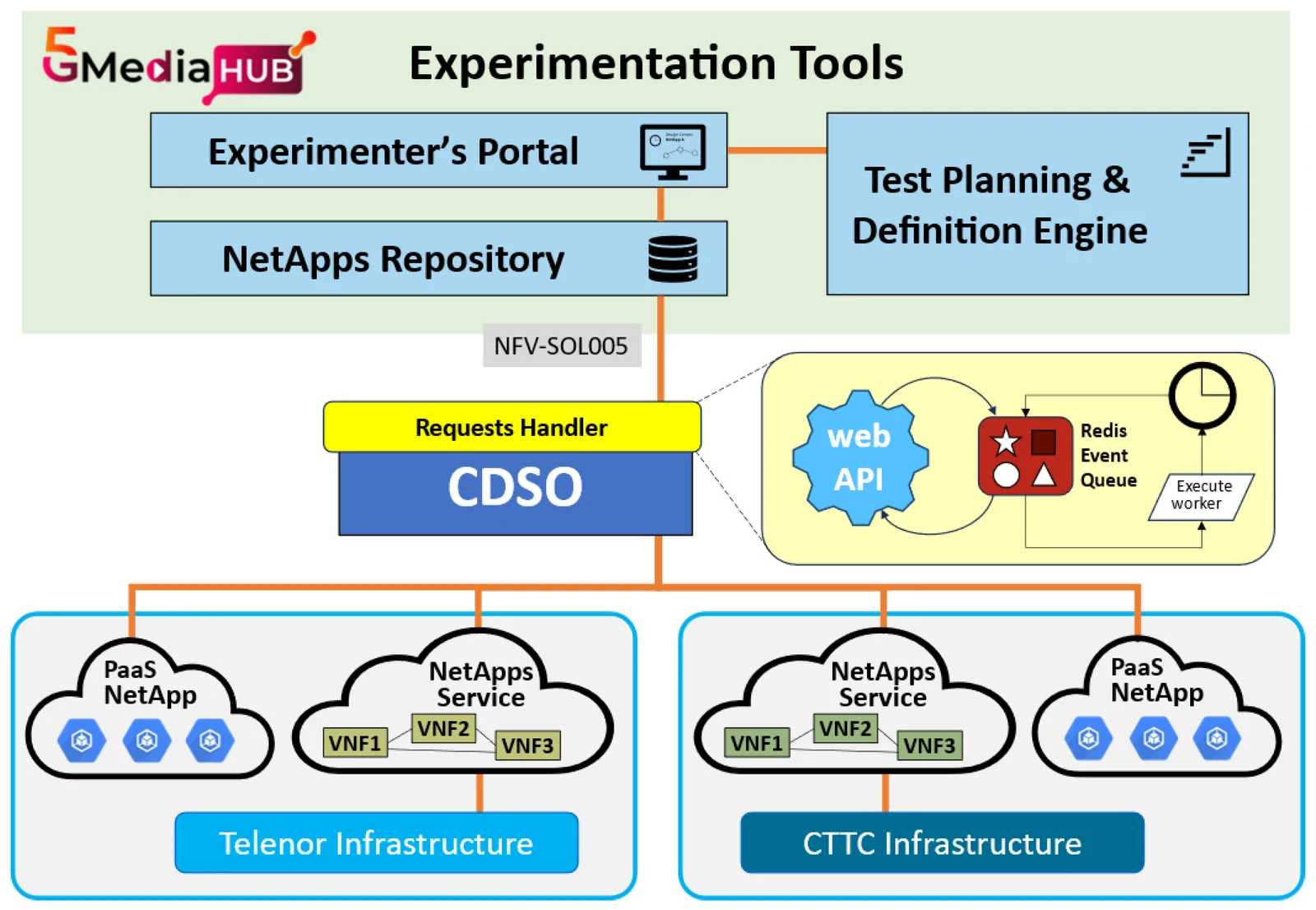
The 5GMediaHUB use case: implementation and integration perspective.

In this section, 5GMediaHUB’s *Experimentation Tools* layer is first described, which acts as the Custom System, followed by the current CDSO Requests Handler implementation and its integration with the NetApps Repository. An illustrative overview of the 5G-MediaHUB use case is presented in Figure 5.

### 5.1. Custom System

The *Experimentation Tools* layer generates all requests regarding NetApp onboarding and experiments, including UE specifications and NetApps, among others. The CDSO will mainly have northbound interactions with the NetApps Repository. In particular, the NetApps Repository application will facilitate the design and onboarding of NetApps to the CDSO.

From the experimentation perspective, a 5GMediaHUB Experimenter can define a test case scenario and indicate the required resources in terms of UEs and NetApps. The Test Planning and Definition Engine will then assess the requested resources and add in the network resources (i.e., 5G NSs or subslices) required to support the experiment. If the experiment is approved—meaning that there are enough resources in the facility and the experiment is compliant to the followed fair-use policy—an *E2E slice request* will be generated. The request will include the (multi-domain) subslice(s) for the NetApps and 5G NSs involved in the scenario, as well as the *UE specification*.

The NetApps implement monitoring interfaces for the test cases and push data to a Data Collector endpoint. All data capture, analysis, and test results are then contextualized in the Experimenter’s Portal, with the real-time collection and storage of KPIs/metrics via the monitoring interfaces.

### 5.2. Requests Handler Implementation

The CDSO’s Requests Handler implemented in 5GMediaHUB utilizes the message broker feature of a Redis database. Redis is an open source (BSD licensed), in-memory data structure store used as a database, cache, message broker, and streaming engine [21]. This serves as a cloud-native backend for a Redis Queue (RQ), which is a simple Python library for queuing jobs/events in a FIFO manner to be processed in the background.

A job or event is a Python object, representing the Requests Handler function that should be invoked as a background process. The RQ can easily be integrated into a web stack (e.g., using a Python Flask server as a web API). It is important to note that as a cloud-native solution, the CDSO is inherently scalable and does not pose a performance bottleneck, since its software components can be dynamically scaled to meet demands.

The internal elements of the Requests Handler are shown in the yellow block in Figure 5. The web API in the current implementation accepts (i) *OSM requests* in relation to either the CDSO’s internal OSM instance for NetApps or an OSM-based 5G SO of a given domain for 5G NSs; and (ii) *PaaS requests* in relation to available PaaS instance(s) from the domain(s). Each request returns a unique 128-bit identifier, which must be used when checking the status of the request, e.g., if it is queuing, executing, or has ended successfully. Requests to other 5G SO implementations (e.g., proprietary solutions such as Nokia’s orchestration stack) can also be easily added once a service ordering API is made available.

### 5.3. NetApps Repository Integration

**Figure 6:**
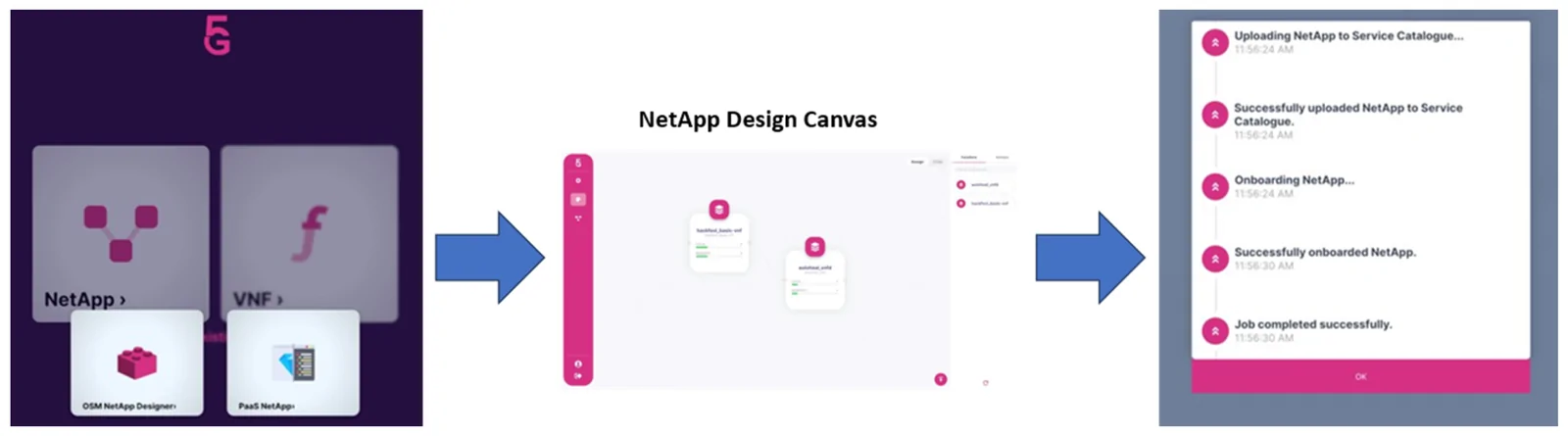
From NetApp design to onboarding.

The NetApps Repository is a multi-component system forming a key part of the *Experimentation Tools*, facilitating the design and onboarding of NetApps via the CDSO. It is intended as an experimentation tool for 5GMediaHUB developers, offering flexible and user-friendly design and the deployment of NetApps, as shown in Figure 6. The NetApps Repository consists of several interconnected components, as follows: (i) a back-end service, which serves as the core of the system; (ii) a front-end web application for user interaction; (iii) a Service Catalogue for function discovery; and (iv) a GitLab instance for NetApp project storage.

All NetApps Repository deployment queries are relayed via the Requests Handler, allowing seamless integration with the CDSO. This enables the NetApps Repository to query/create/update/delete NetApp packages and deploy NetApps at either testbed via a unified interface. A command prefix mechanism ensures that requests are directed to the appropriate endpoint, facilitating multi-testbed operations and both standard Kubernetes and NFV-based NetApp deployments.

### 5.4. Evaluation

The 5GMediaHUB use case mainly focused on the architecture and workflow design, as well as the development of the CDSO and Custom System components, with the aim of delivering a proof-of-concept over the two domains provided by Telenor and CTTC. This involves functional tests for using the CDSO’s Requests Handler to access the VIMs/CISMs, PaaS, and 5G SOs from the *Experimentation Tools* layer.

**Figure 7:**
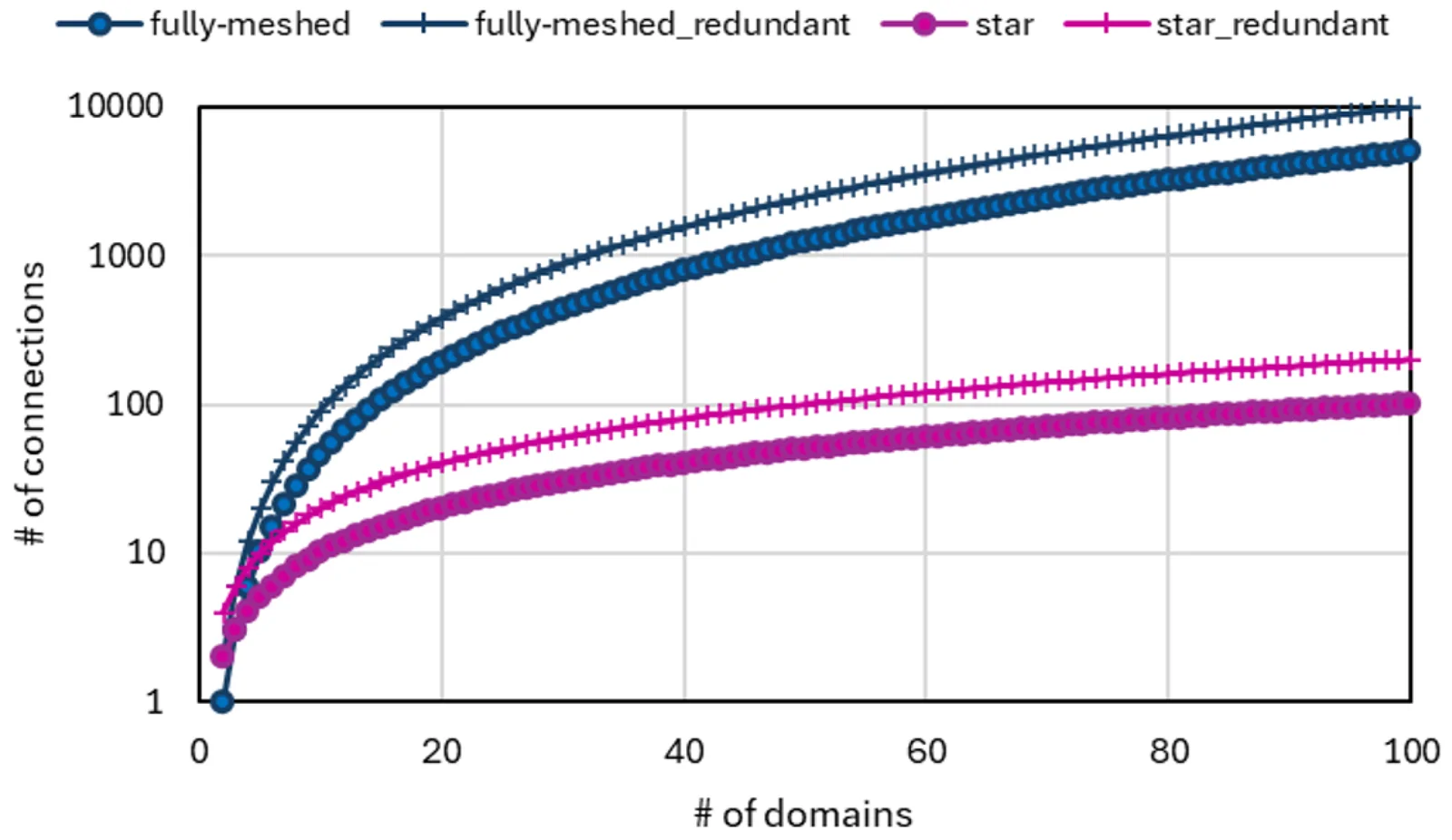
Scalability evaluation.

Moreover, the scalability of the architecture (from a potential fully meshed to a star setup) has been evaluated. As shown in Figure 7, the number of connections grows quadratically (n(n−1)/2) for fully meshed architectures and only linearly (*n*) for a star architecture with the CDSO, where *n* is the number of domains. The complexity of federation is also substantially reduced as individual domains can have their own 5G SO systems, as well as being able to control how many VIM/CISM resources they will expose to the unified CDSO.

## 6. Conclusions

The use of 5G technologies is expected to enable more disruptive services and applications from a wide range of verticals and use case scenarios. However, to ensure technology uptake by SMEs and 3rd party developers, it has become fundamental to abstract the complexities of the underlying infrastructure, platform, and related management and orchestration systems, especially in multi-domain scenarios.

A novel approach for cross-domain service orchestration is proposed, combining the flexibility of supporting different 5G SOs at the platform level, while preserving compatibility at the NetApps level. With ETSI’s OSM as a basis for the CDSO, a Requests Handler module is designed to be developed on top that would act as the main integration point to the different components of the domains’ infrastructure/platform (i.e., 5G SOs and DMSs) and Custom System(s) northbound. This would facilitate the interworking among independently orchestrated domains in supporting multi-domain services. Workflows for NetApp onboarding, E2E slice ordering, and monitoring service ordering are also illustrated and described.

Lastly, the 5GMediaHUB use case for the CDSO is presented, which is a proof-of-concept for the proposed cross-domain orchestration approach that substantially improves scalability and reduces federation complexity. With 5GMediaHUB’s *Experimentation Tools* layer acting as the Custom System, the design, onboarding, and testing of NetApps and next-generation scenarios for the media industry can be simplified and can accelerate the development and rollout of disruptive media services.

## Data Availability

The original contributions presented in this study are included in the article. Further inquiries can be directed to the corresponding author.

## Abbreviations

The following abbreviations are used in this manuscript:

BSSs	Business Support Systems
CDSO	Cross-domain Service Orchestrator
CISM	Container Infrastructure Service Manager
CNF	Cloud-native Network Function
CSMF	Communication Service Management Function
E2E	End-to-End
IM	Information Model
MANO	Management and Orchestration
NBI	Northbound Interface
NetApp	Network Application
NFV	Network Function Virtualization
NFVI	Network Function Virtualization Infrastructure
NS	Network Service
NSMF	Network Slice Management Function
NSSI	Network Slice Subnet Instance
NSSMF	Network Slice Subnet Management Function
OSM	Open Source MANO
OSSs	Operations Support Systems
PaaS	Platform-as-a-Service
PNF	Physical Network Function
QoE	Quality of Experience
QoS	Quality of Service
RQ	Redis Queue
VIM	Virtual Infrastructure Manager
VNF	Virtual Network Function
